# Potential mechanisms and clinical applications of static magnetic field therapy in glioma

**DOI:** 10.3389/fneur.2025.1594874

**Published:** 2025-06-25

**Authors:** Ziyu Sun, Kairui Zhu, Wenxuan Zhao, Xi-feng Fei, Lei Shi, Yong Zhang

**Affiliations:** ^1^Department of Neurosurgery, The First People’s Hospital of Kunshan, Gusu School, Nanjing Medical University, Suzhou, China; ^2^Department of Neurosurgery and Nursing, Affiliated Kunshan Hospital to Jiangsu University, Suzhou, China; ^3^Department of Neurosurgery, Suzhou Kowloon Hospital, Shanghai Jiaotong University School of Medicine, Suzhou, China

**Keywords:** glioma, static magnetic field, non-invasive treatment, apoptosis, tumor microenvironment

## Abstract

Static magnetic field (SMF) therapy, a non-ionizing and non-invasive treatment modality, has garnered increasing attention in glioma research. Gliomas, particularly glioblastoma (GBM), represent one of the most aggressive malignancies of the central nervous system, with limited therapeutic options and significant treatment-related toxicity. Emerging evidence suggests that SMF therapy exerts antitumor effects by inducing apoptosis, inhibiting cell proliferation, and modulating the tumor microenvironment, while minimizing damage to surrounding healthy tissues. Despite promising preclinical findings, research on SMF therapy remains in its early stages, and its precise mechanisms, clinical efficacy, and safety require further elucidation. This review summarizes current advancements in SMF therapy for gliomas, explores its potential as a standalone or adjunctive treatment, and discusses future research directions to optimize its therapeutic application.

## 1 Introduction

Gliomas are among the most aggressive primary malignant tumors of the central nervous system, with glioblastoma (GBM) accounting for approximately 57% of all malignant gliomas (1). Despite advances in therapeutic strategies, the prognosis for GBM remains poor, with a five-year survival rate of less than 5% (1). The current standard of care—comprising maximal safe surgical resection followed by concurrent temozolomide chemotherapy and radiation therapy—extends median survival to only 14–16 months (2). However, this approach is significantly limited by challenges such as the poor penetration of the blood-brain barrier and systemic toxicities, including myelosuppression and cognitive dysfunction (3). These limitations underscore the urgent need for novel, effective, and less toxic therapeutic strategies for glioma treatment.

In recent years, static magnetic field (SMF) therapy has emerged as a promising non-ionizing, non-invasive physical treatment modality (4). Although research on SMF therapy for gliomas remains limited, preliminary *in vitro* and animal studies suggest that SMFs may directly inhibit tumor growth by inducing apoptosis and suppressing cellular proliferation (5, 6), while exerting minimal cytotoxicity on adjacent normal tissues (4). Although research on SMF therapy for gliomas remains limited, existing evidence indicates that SMF may clinically, the non-invasive nature and relatively low side-effect profile of SMF therapy make it an attractive therapeutic option, particularly for patients who are resistant to or intolerant of conventional treatments (7–9).

Despite these promising findings, many aspects of SMF therapy remain to be fully elucidated, including its precise mechanisms of action, optimal treatment parameters, and potential clinical efficacy. Further research is required to establish standardized protocols, evaluate long-term safety, and explore its integration into current glioma treatment paradigms.

This review aims to comprehensively summarize and analyze the current research on SMF therapy for gliomas. Key aspects covered will include the clinical efficacy of this treatment, its potential mechanisms of action, its impact on patients’ quality of life, factors influencing treatment outcomes, the development of predictive models for therapeutic efficacy, and the optimization of treatment strategies. By reviewing advancements in these areas, we hope to provide valuable insights for current clinical practices. Additionally, we will discuss future directions to inspire novel approaches and renewed hope in the treatment of gliomas.

## 2 Clinical efficacy of static magnetic field therapy in gliomas

### 2.1 Short-term efficacy

Recent clinical evidence suggests that static magnetic field (SMF) therapy may hold potential in short-term tumor control for gliomas. One exploratory clinical study evaluated a portable device (Nativis Voyager^®^), which emits ultra-low frequency radiofrequency energy (0–22 kHz), in patients with recurrent glioblastoma. While the study reported no significant device-related adverse events and observed median progression-free survival (PFS) of 10 weeks in the Voyager-only group (*n* = 4) and 16 weeks in the Voyager plus standard-of-care (SoC) group (*n* = 7), it is important to note that the sample size was small and no laboratory data corresponding to specific time points were provided (“data not shown”). The conclusions regarding safety and efficacy were primarily based on imaging (MRI), PFS, and overall survival (OS), with OS ranging from 11 to 16 months (10). These findings are preliminary and should be interpreted with caution until further data from larger and controlled studies become available. A clinical trial involving advanced non-small cell lung cancer (NSCLC) revealed that patients who received daily exposure to 0.4 T static magnetic field (2 h per session, for 6–10 weeks) had a median survival of 6.0 months, significantly longer than the historical control group receiving supportive care (HR = 0.62, *p* < 0.05). Additionally, patient pain scores decreased, and no grade 3 or higher toxicities were reported (11). Although the study was not conducted on central nervous system (CNS) tumors, its findings provide theoretical support for the potential application of static magnetic fields (SMFs) in glioma treatment, as similar antitumor mechanisms—such as inhibition of cell proliferation and modulation of the tumor microenvironment—may be involved. However, caution is warranted when extrapolating results from non-CNS tumors to gliomas. The unique microenvironment and biological characteristics of glioma cells may lead to different responses, indicating that such extrapolations may not always be directly applicable.

### 2.2 Medium-term and long-term efficacy

Currently, large-scale controlled studies are lacking, and it remains uncertain whether static magnetic field (SMF) therapy can significantly extend overall survival in patients with glioblastoma. However, animal studies provide supporting evidence. Novikov et al. (12) investigated the effects of combined weak static (42 μT) and extremely low-frequency alternating magnetic fields (1, 4.4, and 16.5 Hz; 100–300 nT) on mice bearing Ehrlich ascites carcinoma. Their study demonstrated significant tumor suppression, with some tumors exhibiting necrosis and complete disappearance. Importantly, these magnetic field exposures did not induce pathological changes in the organs of healthy mice, suggesting the treatment’s safety. Furthermore, a study by Zhang et al. reported that ultra-low frequency gradient magnetic fields could induce apoptosis in tumor cells and inhibit the formation of new blood vessels, which contributed to the suppression of tumor growth in mice. These findings suggest that prolonged exposure to static magnetic fields may have a tumor-suppressive effect in the medium term (10). Additionally, research using a breast cancer xenograft model observed that long-term daily exposure (6 h per day for 4 weeks) to a magnetic field significantly slowed tumor growth, with the average tumor volume being smaller compared to the non-exposed group (4). Thus, it can be speculated that the application of an appropriate dosage of static magnetic fields over a prolonged period in glioblastoma patients may extend their progression-free survival. However, data from medium-term and long-term follow-up studies in glioblastoma patients are currently very limited, with only a few feasibility studies and case reports available (13).

Overall, the clinical efficacy of static magnetic field therapy in tumor treatment remains under investigation. Although this therapy can stabilize the condition and alleviate symptoms in the short term (11), its potential to significantly extend patient survival requires further validation through additional prospective controlled studies.

## 3 Mechanisms of static magnetic field in glioma treatment

### 3.1 Regulation of cell proliferation and apoptosis

The biophysical effects of static magnetic fields on tumor cells are multifaceted. Several *in vitro* studies have demonstrated that static magnetic fields at appropriate intensities can inhibit the proliferation of glioma cells. They can also induce cell cycle arrest and apoptosis (14, 15). For instance, a study by Zeng et al. (14) showed that exposure to ultra-low frequency gradient static magnetic fields resulted in prominent apoptotic characteristics in tumor tissues of mice, including cell shrinkage, rounded shape, chromatin condensation, and an increased TUNEL-positive rate. Additionally, the tumor DNA content in the magnetic field-treated group decreased, suggesting that the cell cycle was blocked during the synthesis phase, thereby inhibiting cancer cell division (14). Another study reported that exposure to a static magnetic field of 1.4–2.6 T for 48 h reduced the viability of human glioma cells by 20–30%, accompanied by a downregulation of CDK1 expression. The decrease in cell viability was primarily due to the disruption of mitotic spindle formation (15). Furthermore, Sun et al. (16) demonstrated that SMF exposure significantly enhanced apoptosis in glioma cells, even in the presence of TGF-β1, which typically suppresses apoptotic activity. This finding suggests that SMFs may counteract TGF-β1-induced anti-apoptotic effects, offering potential therapeutic benefits in glioma treatment. Therefore, static magnetic fields may inhibit proliferation by inducing cell cycle arrest, and cell death may occur through apoptosis or necrosis, depending on the magnetic field parameters and cell type (15). Overall, the inhibition of tumor cell proliferation and induction of programmed cell death are key mechanisms by which static magnetic fields exert anti-glioma effects.

### 3.2 Effects on molecular signaling pathways

Static magnetic fields regulate cell signaling by altering molecular structures and modulating ion channels. Strong static magnetic fields can directly influence certain proteins. For instance, a study utilized liquid-phase scanning tunneling microscopy to observe purified epidermal growth factor receptor (EGFR), revealing that a static magnetic field of 1–9 T altered the orientation of the EGFR kinase domain in solution and interfered with its dimerization and downstream activation (17). In cellular experiments, exposure to a 9.4 T ultra-strong static magnetic field reduced the proliferation rate of cancer cells overexpressing EGFR by approximately 35%, with minimal effects on control cells lacking EGFR (17). This suggests that static magnetic fields may inhibit signaling pathways by altering the conformation and function of key oncogenic proteins (e.g., EGFR and other receptor kinases) in cancer cells (17). Moreover, static magnetic fields also regulate the function of membrane proteins such as calcium ion channels. Teodori et al. (18) reported that exposure to a 6 mT static magnetic field significantly increased the intracellular free Ca^2+^ concentration in primary glioma cells (from approximately 124 nM to 233 nM) and altered calcium homeostasis. Interestingly, magnetic field exposure significantly reduced the proportion of apoptotic cells induced by heat shock and etoposide, with a reduction of approximately 44–56% (18). The authors hypothesized that the changes in calcium flux induced by the magnetic field may activate protective signaling pathways within the cell, temporarily increasing tolerance to damaging stimuli and thus inhibiting stress-induced apoptosis (18). This finding suggests that the effects of static magnetic fields on cellular signaling are dual in nature: they can promote apoptosis in tumor cells, but under certain conditions, they may protect cells from external damage (a consideration that needs to be addressed in combination therapies).

### 3.3 Tumor microenvironment and blood-brain barrier

Static magnetic fields may exert effects by modulating the tumor microenvironment (19). On one hand, magnetic fields can influence tumor blood supply (20) and angiogenesis (21). Early studies have shown that static magnetic fields inhibit endothelial cell proliferation and migration, reducing tumor angiogenesis, which leads to tumor ischemia and necrosis (4, 11). For instance, it has been observed that magnetic field exposure reduces the vascular density of tumor tissues, which is proposed to be one of the key mechanisms underlying the antitumor effect (4). On the other hand, the impact of static magnetic fields on the blood-brain barrier (BBB) has also garnered considerable attention. The BBB is a major obstacle to drug delivery in glioma therapy (22). Recent studies have investigated the effects of static magnetic fields (SMFs) on the blood-brain barrier (BBB). Yang et al. (23) demonstrated that SMFs generated by MRI scanners can attenuate microbubble cavitation during focused ultrasound (FUS), leading to a significant reduction in BBB opening and decreased delivery of agents like Evans blue dyeca. Conversely, studies have demonstrated that when magnetic nanoparticles are used, their distribution *in vivo* is significantly altered by an external static magnetic field, enabling them to penetrate the BBB and reach brain lesions (24, 25). Moreover, the magnetic field-directed guidance of nanocarriers significantly enhances drug delivery efficiency (24). This strategy suggests that static magnetic fields hold potential for integration with nanomedicine, enabling targeted therapy for gliomas.

In summary, static magnetic fields exert antitumor effects on gliomas through multiple mechanisms: inhibiting tumor cell proliferation and inducing cell death, interfering with oncogenic signaling pathways, altering ion balance and the cytoskeleton, inhibiting tumor angiogenesis, and assisting drug penetration across the BBB into tumor regions. The complexity of these mechanisms suggests selective effects of magnetic fields on different glioma cells and microenvironments, providing a basis for the development of optimized treatment strategies.

## 4 Impact of static magnetic field therapy on patient quality of life

Compared to traditional radiotherapy and chemotherapy, static magnetic field (SMF) therapy offers potential advantages due to its non-invasive nature and absence of ionizing radiation. SMF therapy has been used to alleviate symptoms of diabetic peripheral neuropathy in patients with type 2 diabetes and can improve patients’ quality of life (QoL) (26). Evaluating quality of life (QoL) is particularly crucial in glioma treatment, as the disease profoundly impacts neurological function and daily living. Preliminary clinical observations suggest that static magnetic field (SMF) therapy may alleviate symptoms and help preserve neurological function. Therefore, integrating QoL endpoints into SMF therapy research is essential for a comprehensive understanding of its potential benefits.

### 4.1 Symptom relief

Magnetic field therapy has emerged as a promising adjunctive treatment in clinical practice due to its lack of significant toxic side effects (27–29). Studies have shown that it not only avoids toxicity but also significantly alleviates systemic symptoms and cancer-related pain in advanced lung cancer patients (11). While the study participants were not glioma patients, the findings suggest that magnetic field therapy may help alleviate tumor-related symptoms and improve patient comfort (11). While there is currently no direct clinical evidence that static magnetic field (SMF) therapy alleviates symptoms caused by tumor-induced intracranial pressure in glioma patients, such as headaches, nausea, or neurological deficits, this hypothesis is proposed based on the known pathophysiology of glioma-related mass effects. Further research is necessary to investigate this potential therapeutic benefit.

### 4.2 Neurocognitive function

Gliomas and their treatments frequently impair cognitive and motor functions (30–32). Static magnetic fields do not directly damage neurons, nor do they tend to cause cognitive dysfunctions as chemotherapy drugs often do. Patients receiving magnetic field therapy are more likely to maintain cognitive and motor functions when their condition is stable. A study on recurrent glioblastoma patients using the Voyager device showed no reports of neurofunctional deterioration following treatment, suggesting that this therapy is both safe and well-tolerated (10). Additionally, studies conducted on healthy volunteers using transcranial static magnetic stimulation (tSMS) revealed that brief exposure to magnetic fields reversibly reduced motor cortex excitability without affecting cognitive function (33). While these findings suggest that short-term SMF exposure may have mild and reversible effects, current evidence remains limited, and further research is needed to conclusively determine the long-term impact of SMF therapy on cognitive and neurological functions in glioma patients.

### 4.3 Social, psychological, and adherence factors

Static magnetic field therapy typically involves wearable or bedside devices. This treatment does not require hospitalization or complex care, making it easier for patients to integrate it into their daily lives and reducing the burden of frequent hospital visits. A portable static magnetic field device designed to be worn on the head is user-friendly, allowing patients to self-administer the therapy at home. Studies have shown that this device minimally interferes with daily activities and has good patient adherence (10), suggesting that patients can maintain social participation and psychological well-being during long-term treatment without being disrupted by complex procedures. In contrast, severe bone marrow suppression caused by chemotherapy or brain damage induced by radiation therapy often leads to treatment interruptions and a dramatic decline in quality of life. The safety of static magnetic field therapy offers glioma patients a low-burden, high-tolerance alternative that could extend high-quality survival time.

It is important to note that current understanding of how static magnetic fields improve the quality of life in glioma patients is primarily based on observational data regarding symptoms and functionality, due to the lack of quantified data on quality of life. Future clinical trials should incorporate quality of life assessments using scales such as the EORTC QLQ-C30 or BN20 to systematically monitor cognitive function, emotional state, and social adaptation, providing a more comprehensive understanding of the benefits of magnetic field therapy. Based on the current evidence, it can be cautiously concluded that static magnetic field therapy is unlikely to significantly impair the quality of life in glioma patients. On the contrary, it may provide benefits through symptom management and reduced side effects (10, 11). Therefore, static magnetic field therapy offers glioma patients a new treatment option that not only effectively controls tumor progression but also preserves quality of life to a significant extent. With further research, future studies may optimize treatment protocols and enhance patient adherence and overall well-being.

## 5 Factors influencing the effectiveness of static magnetic field therapy

The effectiveness of static magnetic field therapy varies among individuals and experiments, owing to multiple factors, including magnetic field parameters, tumor biological characteristics, and patient conditions.

### 5.1 Magnetic field intensity and frequency

The physical parameters of the magnetic field are crucial determinants of therapeutic efficacy. Research has demonstrated that the inhibitory effects of static magnetic fields on cell proliferation are intensity-dependent and frequency-dependent (17). Generally, higher magnetic field intensities produce more pronounced biological effects. For instance, studies have shown that in cells with overexpressed EGFR, increasing magnetic field intensities (within the 1–9 T range) led to more significant conformational changes in EGFR and stronger inhibition of cell proliferation (17). Conversely, magnetic fields with very low intensities (geomagnetic fields of 50 μT) may have weak and barely detectable effects. While this review focuses on static magnetic fields, studies on extremely low-frequency (ELF) magnetic fields also suggest the importance of frequency parameters. For example, a 50 Hz magnetic field exhibited different effects compared to 100 Hz in some combination therapies (34). Therefore, it is essential to optimize the intensity and frequency of the magnetic field when designing therapeutic protocols. Excessively high magnetic fields may pose safety risks or introduce device complexity, while overly weak fields may be ineffective. Current clinical devices typically use moderate intensity (ranging from hundreds of milliteslas to several teslas) and static or low-frequency magnetic fields to balance therapeutic efficacy with safety (15). For instance, clinical trials using a 0.4 T static magnetic field for daily irradiation of brain tumor regions have shown some therapeutic benefits (11). Further dose-response studies are needed to determine the optimal magnetic field dosage.

### 5.2 Tumor type and molecular characteristics

Different glioma types and subtypes may respond differently to magnetic fields. *In vitro* studies comparing the magnetic field sensitivity of various cell lines have identified cell type as a critical factor influencing outcomes (17). For example, tumor cells with highly activated EGFR are more sensitive to strong static magnetic fields, exhibiting significant proliferation inhibition, while normal cells or EGFR-low-expressing cells are unaffected (17). This suggests that the molecular characteristics of the tumor (such as driver gene status) may modulate the effects of magnetic field therapy. In gliomas, molecular markers such as IDH mutations and MGMT promoter methylation are associated with differences in tumor proliferation and resistance, potentially impacting the efficacy of magnetic field therapy. Additionally, tumor grading and histological type may also play a role. Low-grade glioma cells, which proliferate slowly, are less responsive to the field’s mechanism of mitotic inhibition, while rapidly proliferating GBM cells may be more sensitive to magnetic fields due to their more frequent mitosis, which is more susceptible to interference. Interestingly, a study comparing the responses of primary glioma cells from four patients to a 6 mT static magnetic field revealed apoptosis reduction ranging from 28 to 87% (18). This inter-patient variability indicates that the intrinsic genetic and epigenetic backgrounds of tumors significantly affect the therapeutic outcomes of magnetic fields (18). Therefore, in clinical practice, the biological characteristics of a patient’s tumor (such as proliferation index and molecular subtype) may determine their response to static magnetic field therapy. Future research may identify biomarkers to screen for patients who are more likely to benefit from magnetic field therapy, thereby improving overall efficacy.

### 5.3 Patient age and health status

The overall health and immune status of patients can also influence the effectiveness of static magnetic field therapy. Older glioma patients tend to have reduced immune function, with weaker immune surveillance within the tumor microenvironment. As a result, any immune-related anti-tumor effects induced by the magnetic field may be less pronounced in these patients. In contrast, younger patients typically exhibit faster metabolism and higher cell proliferation rates, making them more susceptible to the inhibitory effects of magnetic fields on rapidly dividing tumor cells. Furthermore, patient adherence to magnetic field therapy is related to their physical condition. Patients who are frail or suffer from cognitive impairments may find it challenging to comply with long-term home-based magnetic field treatment, thereby indirectly affecting its effectiveness. Magnetic field therapy could provide a beneficial adjunct for patients who cannot tolerate conventional chemotherapy or radiotherapy (e.g., elderly or frail individuals) (11). For example, in the aforementioned lung cancer study, many patients had no standard treatment options available prior to magnetic field therapy, but the median survival for these patients reached 6 months, which was comparable to the outcomes of chemotherapy (11). The patient’s treatment history and disease stage are crucial factors. Advanced, widely disseminated tumors may not respond as well to localized magnetic fields as localized lesions, which can receive sufficient magnetic field doses. Moreover, tumor volume may affect the therapeutic outcome, with smaller tumors being more easily covered by a uniform magnetic field.

### 5.4 Treatment parameters and protocol design

In addition to the aforementioned factors, therapeutic efficacy also depends on the specific implementation of the magnetic field therapy protocol. For instance, it remains unclear whether continuous long-duration exposure is more effective or if intermittent exposure is better suited to inducing cellular stress. Some animal studies have used exposure durations ranging from hours to days (4, 35), while clinical feasibility trials often involve patients wearing devices during waking hours and removing them during rest (10). Whether this time pattern is optimal remains to be determined. Additionally, the spatial gradient distribution of the magnetic field may influence therapeutic outcomes, with reports suggesting that the magnetic field gradient itself can affect glioma cell movement and proliferation (36). Therefore, when designing treatment devices, optimizing the uniformity and gradient of the magnetic field within the tumor region is necessary to enhance therapeutic efficacy. Furthermore, the order of administration in combination therapies, whether synchronous or sequential, may also impact the effects of magnetic field therapy. These parameters need to be clarified through systematic research.

In summary, static magnetic field therapy for gliomas is not a “one-size-fits-all” approach. Its efficacy depends on the matching of physical dosage, tumor biology, and patient conditions. Future research should involve more refined experimental and clinical data to develop predictive models or decision trees that integrate these factors, enabling the personalized design of magnetic field therapy protocols to maximize its therapeutic potential.

## 6 Efficacy prediction models and treatment evaluation

Static magnetic field therapy for gliomas is an emerging field, and currently, the related efficacy prediction models are still in their early stages. The survival analysis and prognostic models commonly used in oncology can be leveraged to assess the effectiveness of static magnetic field therapy and explore corresponding predictive indicator systems.

### 6.1 Clinical outcome evaluation

The first step is to define the key efficacy endpoints for static magnetic field therapy, which include radiological tumor response (such as changes in tumor volume measured by MRI), progression-free survival (PFS), and overall survival (OS). In small-scale clinical studies, these endpoints have been used to preliminarily assess therapeutic effects. For example, trial reports using the Voyager device have presented median PFS and OS to describe treatment outcomes (10). Future studies could compare survival curves between the magnetic field treatment group and the control group, calculating hazard ratios and confidence intervals to quantify efficacy. If possible, randomized controlled trials (RCTs) are recommended to obtain higher levels of evidence. Kaplan–Meier survival analysis and Cox regression models can be employed to assess the independent effect of static magnetic field therapy, with forest plots used to illustrate efficacy differences among subgroups, identifying potential benefit populations.

### 6.2 Prediction model construction

Once patient follow-up data is accumulated, prediction models can be developed to estimate the prognosis of patients undergoing static magnetic field therapy. A nomogram model could be constructed incorporating clinical and biological features, including variables such as patient age, tumor grade, MGMT methylation status, extent of surgical resection, and static magnetic field therapy, to predict 1-year and 2-year survival rates. Internal and external validation should be performed, with the *C*-index used to measure the model’s discriminative ability. A *C*-index of 0.7 or higher suggests that the model has predictive capacity. Furthermore, time-dependent receiver operating characteristic (ROC) curves can be used to calculate the area under the curve (AUC) at specific time points. If the inclusion of “static magnetic field therapy” improves the *C*-index or AUC, it indicates that the therapy significantly contributes to prognosis prediction.

### 6.3 Imaging and biomarkers

To predict efficacy earlier and more accurately, imaging techniques and molecular biomarkers can be explored. Functional MRI, magnetic resonance spectroscopy, and other imaging technologies can monitor tumor metabolism and microenvironmental changes, serving as early predictive indicators of treatment efficacy (36). For instance, MRI has shown promising potential in monitoring changes in the tumor microenvironment, especially in evaluating responses to anti-tumor therapies. Studies have demonstrated that diffusion-weighted MRI (DW-MRI) and dynamic contrast-enhanced MRI (DCE-MRI) can detect early changes in the diffusion of water molecules within tumor cells, making them potential biomarkers for treatment response (37, 38). Specifically, certain studies have shown that the apparent diffusion coefficient (ADC) of tumors significantly changes after treatment, reflecting therapeutic effects earlier than traditional tumor volume assessments. For example, a study found that low ADC values are associated with immune activation in the tumor microenvironment, and MRI characteristics can serve as early biomarkers of immune responses in HER2-positive breast cancer patients (39, 40). Moreover, integrating machine learning algorithms can further combine these multi-dimensional data to enhance predictive accuracy regarding patient treatment responses, thereby providing valuable support for clinical decision-making (41, 42). In summary, the combination of imaging technologies and biomarkers provides new perspectives and tools for early prediction of treatment responses, and future research should continue to explore their clinical application potential to enable more precise individualized therapies.

### 6.4 Model validation and optimization

It is important to note that the available data is still limited. As such, any predictive model should be approached with caution to avoid overfitting and bias. During model development, a portion of the data should be set aside for independent validation, and cross-validation should be employed to assess the robustness of the model. As more prospective trial results are published, the model can be continuously updated and iterated. Furthermore, the predictive model for static magnetic field therapy should be compared with existing prognostic scores for gliomas (such as the RPA classification and the EORTC prognostic model) to assess its added value. If it is found that the efficacy prediction of static magnetic field therapy requires specific parameters (e.g., magnetic field dosage), a specialized prediction system should be developed. Ultimately, these models aim to estimate a patient’s potential benefit prior to the initiation of treatment, assisting physicians and patients in making informed decisions about whether to attempt static magnetic field therapy and whether it should be combined with other therapeutic modalities to achieve the optimal outcome.

In conclusion, the application of efficacy prediction models will make static magnetic field therapy more precise and personalized. At this stage, efforts should focus on collecting standardized clinical follow-up data to lay the foundation for the development of reliable predictive models. Until these models are fully mature, comprehensive efficacy assessments (including regular imaging reviews and quality-of-life evaluations) for each patient undergoing static magnetic field therapy remain essential to timely adjust treatment strategies.

## 7 Optimization and future directions in treatment strategies

To maximize the therapeutic effect of static magnetic field therapy in gliomas, future research must focus on optimizing treatment modalities, combination strategies, and technological approaches. Several key areas warrant attention.

### 7.1 Combination therapy strategies

Static magnetic field therapy is likely to achieve its optimal effect when combined with existing standard treatments. Combination chemotherapy is a crucial approach, but careful design is necessary to avoid potential interference between different treatments. Current studies have shown that the effect of magnetic fields on chemotherapy drugs is highly dependent on the frequency and intensity. For instance, a 50 Hz, 70 G magnetic field was found to reduce the cytotoxicity of carboplatin on U87 glioma cells, whereas a 100 Hz, 100 G magnetic field enhanced the cytotoxic effect of temozolomide (34). This finding emphasizes that combinations of different drugs with magnetic fields may lead to either synergistic or antagonistic effects. Therefore, optimizing combinations is particularly important when designing combination therapy protocols.

Future research should focus on screening the interactions between magnetic fields and chemotherapy drugs through *in vitro* experiments to identify effective synergistic combinations. For example, it might be worth exploring the combination of temozolomide with specific magnetic field parameters, or administering carboplatin in a staggered manner to maximize therapeutic efficacy (34). Additionally, studies combining radiation therapy are equally important, as magnetic fields may enhance the effects of radiotherapy by altering cellular sensitivity to radiation, increasing intracellular reactive oxygen species (ROS) levels, thereby improving tumor responses to radiation therapy while protecting normal cells from radiation-induced damage (43, 44).

Combination immunotherapy also represents an emerging frontier. Studies suggest that magnetic fields may enhance the infiltration of immune cells, and when combined with immunotherapeutic agents, could produce a synergistic anti-tumor effect. The specific combination timing and dosing will need to be optimized through experimental trials to ensure the best treatment outcomes (45, 46). Overall, static magnetic field therapy, as an emerging modality, holds significant potential in combination therapies and warrants further in-depth investigation.

### 7.2 Optimization of magnetic field parameters

Static magnetic fields (SMFs) have shown potential in glioma therapy, with studies indicating that specific parameters—such as field strength, exposure duration, and orientation—can influence therapeutic outcomes. For instance, Hambarde et al. (35) demonstrated that spinning oscillating magnetic fields (sOMF) effectively induce reactive oxygen species (ROS) in glioma cells, leading to selective cytotoxicity without harming normal cells. Their findings emphasize the importance of precise control over magnetic field configurations to maximize therapeutic efficacy. Additionally, research by Mozhdehbakhsh Mofrad et al. (47) on magnetic fluid hyperthermia (MFH) for glioblastoma treatment highlighted that MFH is more sensitive to frequency changes than to magnetic field strength, underscoring the need for careful optimization of these parameters to enhance treatment outcomes while minimizing damage to healthy tissue.

For instance, animal studies utilizing a 21.1 T ultra-high-field magnet, for brief treatment periods, have preliminarily demonstrated its efficacy and safety for tumor treatment (48). Magnetic hyperthermia (MHT), using superparamagnetic iron oxide nanoparticles (SPIONs) in combination with alternating magnetic fields (AMF), has shown potential in inhibiting tumor growth. Studies suggest that at appropriate magnetic field intensities and frequencies, SPIONs can effectively raise the temperature of tumor cells to 42–43°C, thereby inducing apoptosis and significantly inhibiting tumor metastasis and growth (49, 50). Additionally, research has found that the polarity and direction of the magnetic field also influence treatment outcomes. Future studies could consider the design of rotating magnetic fields to prevent cellular adaptation to the magnetic field (51). Further exploration could focus on “sequential magnetic field therapy,” where ultra-strong static magnetic fields are applied during or after tumor resection to eliminate residual tumor cells, followed by conventional magnetic fields for maintenance treatment. This approach may not only improve therapeutic efficacy but may also reduce the risk of postoperative recurrence (52). In this field, leveraging the selective effects of magnetic fields on tumor cells, combined with advanced technologies such as bioinformatics and artificial intelligence, may offer new perspectives for personalized treatment strategies (53, 54). In conclusion, optimization of magnetic field therapy will provide new directions for future cancer treatment, and the combination of various therapeutic strategies and techniques holds promise for achieving more precise and effective tumor management.

### 7.3 Technological and equipment advancements

In order to better apply static magnetic field therapy to gliomas, innovations in equipment and drug delivery methods are required. One promising direction is transcranial magnetic field focusing technology. Current head-based magnetic therapy devices typically generate a uniform magnetic field, making it challenging to focus the field on deep-seated tumors. Drawing inspiration from stereotactic techniques in radiosurgery, the development of directional magnetic field generators is warranted. For instance, a multi-coil array could be employed on the head, with coil currents adjusted based on tumor location, resulting in enhanced field strength in the tumor region and reduced field strength in surrounding tissues (36). This concept is similar to tumor treating fields (TTFields) used with electrode headgear, but applied to static magnetic fields instead. Additionally, implantable magnetic nanoparticle technology is rapidly advancing. These nanoparticles can not only directly damage tumor cells through static magnetic fields but can also serve as drug carriers to increase local drug concentration while reducing systemic toxicity (55). Recent studies have demonstrated that drug-loaded magnetic liposomal nanoparticles, under the guidance of static magnetic fields, can more efficiently cross the blood-brain barrier and diffuse into glioma spheroid models (24, 56). This represents the early stages of “magnetic targeting therapy.” For postoperative patients, a promising strategy could involve implanting a magnetic source within the surgical cavity to continuously generate a localized strong magnetic field, thereby inhibiting the growth of residual tumor cells. However, the safety of this method still requires further evaluation.

### 7.4 Postoperative rehabilitation and follow-up

The integration of static magnetic field therapy into the comprehensive treatment process for gliomas during postoperative rehabilitation and follow-up requires careful management. Introducing magnetic field therapy early in the postoperative phase can capitalize on the transient window of increased blood-brain barrier permeability, helping to suppress tumor proliferation. The medical team should closely monitor treatment efficacy and side effects, regularly assessing tumor control via MRI and conducting neurocognitive tests. If tumor progression is detected, treatment plans should be adjusted accordingly. Magnetic field therapy requires multidisciplinary collaboration, with a focus on oncological markers and patient recovery. Ideally, magnetic field therapy can extend the period of tumor stability, providing patients with more time for recovery and social activities. Thus, the future direction involves integrating magnetic field therapy into a patient-centered management framework, with the development of standardized procedures through multidisciplinary coordination.

### 7.5 Safety improvements

Although static magnetic field therapy has demonstrated good safety in the treatment of gliomas, it is crucial to address the potential risks associated with stronger magnetic fields and various combination therapies in future applications. Extremely strong magnetic fields may not only interfere with implanted medical devices, leading to functional failure, but could also have adverse effects on the patient’s auditory and balance systems. Therefore, establishing a comprehensive contraindication screening process is essential to ensure that patients fully understand the potential risks before undergoing treatment and receive appropriate educational guidance. Long-term safety monitoring is key to ensuring the safe application of static magnetic field therapy and its continued effectiveness. While no serious adverse events have been reported to date, the chronic effects on normal brain tissue warrant further investigation. As technology advances, the implementation of precise magnetic field control systems and real-time dose monitoring technologies will help enhance the safety and effectiveness of treatment.

To address these concerns, cross-disciplinary innovation is key to optimizing static magnetic field therapy for gliomas. The integration of biological mechanisms with engineering technologies will provide new perspectives for adjusting therapeutic strategies, assisting researchers in identifying the optimal application methods. Over the next 5 to 10 years, large-scale randomized controlled trials are expected to be conducted to validate the effectiveness of static magnetic field therapy and promote the development of next-generation intelligent magnetic field therapy devices, further enhancing the safety and effectiveness of treatments.

## 8 Conclusion and outlook

This review systematically summarizes the research progress in static magnetic field therapy for gliomas over the past two decades. Static magnetic field therapy has shown potential as an adjunctive treatment for gliomas overall. Both *in vitro* and animal studies consistently demonstrate its ability to inhibit tumor cell proliferation, induce apoptosis, and suppress angiogenesis. Preliminary clinical studies indicate that it is safe and feasible, with some patients showing symptom improvement and survival benefits. Static magnetic field therapy operates through multiple mechanisms, including interference with oncogenic signaling pathways (such as EGFR), alteration of ionic environments and the cytoskeleton, and enhancing the tumor cells’ sensitivity to other treatments, all while avoiding additional toxicity. In terms of patient quality of life, the non-invasive nature and mild side effects of this therapy result in good patient adherence, allowing for sustained functional status over extended periods.

While we acknowledge that current evidence remains largely speculative, primarily derived from small-scale clinical trials and laboratory studies, several challenges must be addressed before static magnetic field (SMF) therapy can be widely adopted in clinical practice. First, the stability and significance of its therapeutic effects need to be validated in large-scale clinical trials, with a clear assessment of its added benefit over current standard treatments. Second, further optimization of treatment parameters and protocols is needed to address the needs of different glioma patient groups. Third, in-depth mechanistic studies are required to identify biomarkers that predict therapeutic efficacy, allowing for patient selection and treatment adjustment. Lastly, the integration of static magnetic field therapy with surgery, radiation therapy, chemotherapy, and novel immunotherapies will be crucial for achieving optimal outcomes.

Future research should focus on multi-center clinical trials to evaluate the efficacy and safety of static magnetic field therapy in larger patient populations. For instance, randomized controlled studies incorporating static magnetic field therapy in the postoperative adjuvant therapy stage could assess its impact on progression-free survival. Additionally, interdisciplinary collaboration will be key in advancing this field. By combining knowledge from biomedical engineering, physics, and clinical medicine, the development of novel magnetic field application devices could improve the controllability and precision of the treatment. Given the heterogeneity of gliomas, personalized treatment plans will become especially important, requiring a deeper understanding of how different glioma subtypes respond to static magnetic field therapy.

In the era of precision medicine, static magnetic field therapy offers a novel approach and tool for glioma treatment. Looking ahead, as research progresses, static magnetic field therapy is expected to complement other therapeutic modalities to establish a more comprehensive glioma treatment system, ultimately improving patient survival rates and quality of life. We anticipate that in the next two decades, static magnetic field therapy will transition from laboratory research to clinical practice, marking a significant milestone in glioma treatment.

In conclusion, while static magnetic field therapy shows promising potential in the treatment of gliomas, several challenges remain for its clinical application. These include validating its long-term efficacy, optimizing treatment protocols, and enhancing mechanistic research. Through interdisciplinary collaboration and in-depth scientific exploration, static magnetic field therapy is poised to become a valuable adjunct in the treatment of gliomas.
